# Heat-treated *Lactiplantibacillus plantarum* MYO Improves Muscle Strength and Modulates Gut Microbiota in Older Adults with Reduced Muscle Strength

**DOI:** 10.4014/jmb.2607.07020

**Published:** 2026-10-07

**Authors:** Jin Joo Lee, Ji-Won Song, Hyeon Jeong Kim, Jung Hoon In, Jisoo Park, Jongsun Park, Seunghun Lee

**Affiliations:** 1 Biohealthcare R&D Center, HYUNDAI BIOLAND Co., Ltd., Ansan 15407, Republic of Korea; 2 Mitos Biomedical Institute, Mitos Therapeutics Inc, Daejeon 34134, Republic of Korea; 3 Department of Pharmacology, College of Medicine, Chungnam National University, Daejeon 35015, Republic of Korea; 4 Department of Medical Science, Metabolic Syndrome and Cell Signaling Laboratory, Institute for Cancer Research, College of Medicine, Chungnam National University, Daejeon 35015, Republic of Korea

**Keywords:** Muscle strength, Gut microbiota, Heat-treated *Lactiplantibacillus plantarum*

## Abstract

Age-related sarcopenia is a condition characterized by a decline in muscle strength and physical function with aging, and its improvement is important given its close association with falls, fractures, and mortality. Recently, as the link between age-related gut microbiota dysbiosis and sarcopenia has been elucidated, modulation of the gut microbiota has gained attention as a therapeutic strategy for sarcopenia. In particular, with growing interest in paraprobiotics, various health-promoting effects—including gut microbiota modulation and improvement of muscle strength—have been reported. However, clinical evidence supporting the association between the gut microbiota and muscle strength remains limited. Accordingly, this clinical study was conducted to investigate the effects of heat-treated *Lactiplantibacillus plantarum* MYO (LPMYO) intake on quadriceps muscle strength, hand grip strength, muscle quality index (MQI) and gut microbiota composition in older adults with reduced muscle strength, and to examine the correlation between muscle strength and gut microbiota. After 12 weeks of heat-treated LPMYO supplementation, quadriceps muscle strength, hand grip strength and MQI were significantly improved compared with the placebo group. In addition, the abundance of beneficial bacteria, including *Faecalibacterium*, *Lachnospira*, *Turicibacter*, *Megasphaera*, and the [*Eubacterium*]_*ventriosum* group, was significantly increased. Notably, *Turicibacter* and *Megasphaera* showed positive correlations with muscle parameters. In addition, enrichment of predicted microbial functions, such as amino acid metabolism, nicotinate and nicotinamide metabolism, and oxidative phosphorylation (OXPHOS), was observed following supplementation with heat-treated LPMYO. Taken together, heat-treated LPMYO significantly improved muscle strength and muscle quality, and these muscle strength–enhancing effects may be associated with alterations in the gut microbiota.

## Introduction

The rapid aging of the global population has led to increased attention on sarcopenia as a key determinant of quality of life in older adults [1]. Sarcopenia is characterized by the progressive and systemic loss of skeletal muscle mass, accompanied by declines in muscle strength and physical performance, and is associated with an increased risk of falls, fractures, metabolic disorders, and mortality [2-3]. Therefore, maintaining and improving muscle strength and function is essential for preserving independence and overall health in the elderly.

The gut microbiota, a complex microbial ecosystem residing in the gastrointestinal tract, has been reported to influence not only gut health but also overall health through various mechanisms, including immune regulation, modulation of inflammatory responses, and maintenance of metabolic homeostasis [4]. Dysbiosis of the gut microbiota is considered a hallmark of aging [5]. Recent studies have shown that age-related dysbiosis of the gut microbiota is closely associated with muscle loss and functional decline [6-7]. These alterations are accompanied by changes in microbial-derived metabolites such as short-chain fatty acids (SCFAs), and may influence muscle homeostasis by promoting chronic systemic inflammation and inducing impaired insulin sensitivity and increased anabolic resistance [8-10]. Accordingly, the concept of the gut–muscle axis has emerged, and modulation of the gut environment is considered a promising strategy for improving muscle strength [11-12]. For instance, Chen *et al*. (2025) reported that administration of *Bifidobacterium animalis* subsp. *lactis* A6 increased butyrate-producing bacteria and alleviated muscle loss in a mouse model of dysbiosis-associated muscle wasting [13]. In addition, supplementation with *Lactiplantibacillus plantarum* TWK10 in mice has been shown to improve gut microbial composition and metabolic profiles, thereby attenuating age-related declines in muscle strength [14]. Collectively, these findings suggest that modulation of the gut microbiota may play a critical role in mitigating sarcopenia.

Recent studies have demonstrated that paraprobiotics can also influence the improvement of the gut microbiota [15-17]. Paraprobiotics are defined as inactivated or non-viable microbial cells, or their components, that confer health benefits to the host when administered in adequate amounts [18]. Paraprobiotics contain various bioactive substances, including cell wall components such as peptidoglycan and teichoic acid, as well as polysaccharides, proteins, and lipids, and exert beneficial physiological effects such as strengthening the intestinal barrier and immunomodulation [19-20]. In addition, they have been reported to provide diverse health-promoting effects, including improvement of bowel function, anticancer activity, and alleviation of ulcerative colitis [21-23]. Accordingly, growing attention has been paid to paraprobiotics as an alternative that retains the functional benefits of probiotics while overcoming limitations associated with stability during storage and safety [24]. In this context, growing evidence has highlighted the potential role of paraprobiotics in improving muscle health. Previous studies have demonstrated that supplementation with heat-killed *L. plantarum* TWK10, *B. breve* B-3, and pasteurized *Akkermansia muciniphila* HB05 can enhance muscle mass and improve muscle strength [25-27]. In particular, intake of heat-killed *L. plantarum* TWK10 has been reported to be associated with alterations in gut microbiota composition and microbial metabolic functions [28]. These findings suggest that paraprobiotics may exert muscle strength enhancing effects mediated by the gut–muscle axis.

In our previous study, heat-treated *Lactiplantibacillus plantarum* MYO (LPMYO) inhibited sarcopenic factors including Yin Yang 1 (YY1), a key transcriptional repressor of myoblast differentiation, and promoted myogenesis in dexamethasone-induced C2C12 cells and C57BL/6 mice muscle atrophy models, thereby enhancing myoblast fusion and muscle strength [29]. Based on these findings, this study aimed to investigate the effects of heat-treated LPMYO supplementation on gut microbiota composition and muscle health in elderly individuals with reduced muscle strength, and to examine the association between gut microbiota profiles and muscle-related parameters.

## Materials and Methods

### Study Design

This study was designed as a randomized, placebo-controlled, double-blind, parallel clinical trial. At Visit 1, the eligibility for inclusion and exclusion criteria was assessed only for participants who voluntarily provided informed consent. Participants who met the inclusion criteria were randomly assigned at Visit 2 (Week 0) to either the heat-treated LPMYO culture group (LPMYO) or the placebo group (PLA). Each participant consumed one capsule of the heat-treated LPMYO culture or placebo supplement once daily for 12 weeks. During the entire study period, participants visited the laboratory a total of three times (Weeks 0, 6, and 12). Assessments, including quadriceps muscle strength, hand grip strength, gut microbiota, blood and urine biomarkers, and adverse events, were conducted at Visit 2 (Week 0) and Visit 4 (Week 12). Fecal samples were collected at Weeks 0 and 12 using the AccuStool kit (AccuGene, Republic of Korea) containing a preservative solution and were stored at - 80°C until analysis (Fig. 1). This study was conducted in accordance with the Declaration of Helsinki with approval from the Institutional Review Board (IRB) of Bestian Seoul Hospital (IRB Approval No.: BMC IRB 2025-02-002) following a thorough review of the study protocol.

### Participants

A total of 100 adults aged 50–85 years with reduced muscle strength, but not meeting the diagnostic criteria for sarcopenia, were recruited for this study. The inclusion criteria were as follows: 1) reduced hand grip strength below the standard reference values while not meeting the diagnostic criteria for sarcopenia according to the 2019 Asian Working Group for Sarcopenia (AWGS) guidelines, 2) skeletal muscle mass below 100% of the standard range, and 3) voluntary written informed consent to participate in this clinical trial. The exclusion criteria included individuals with a total score of 4 or higher on the Sarcopenia Quality of Life (SARC-F) questionnaire, those diagnosed with sarcopenia or having a history of fragility hip fractures, and those taking medications related to depression, dementia, or alcoholism. Additionally, individuals with diabetes, hypertension, lung disease, or a history of systemic autoimmune diseases were excluded. Those who had participated in other clinical trials within the past 3 months or had plans to participate in one were also excluded. Detailed exclusion criteria are presented in Table S1.

### Randomization and Blinding

Participants who met the inclusion and exclusion criteria were assigned a number at Visit 2 according to their order of enrollment. They were then randomly assigned in a 1:1 ratio to either the LPMYO or PLA group at the same visit. A double-blind design was employed to ensure that both participants and researchers remained unaware of the group assignments until the conclusion of the study.

### Interventions

From Visit 2 to Visit 4, each participant consumed 450 mg of either the heat-treated LPMYO culture or a placebo once daily for 12 weeks. The LPMYO supplement consisted of 1.8 × 10^11^cells/100 mg of heat-treated LPMYO culture and 350 mg of starch, while the placebo consisted of 450 mg of starch.

### Measurement of Muscle Parameters

To evaluate the improvement in muscle strength, quadriceps muscle strength and hand grip strength (GRIP) were measured pre- (baseline) and post- (endline) intake (Visit 2 and Visit 4). Quadriceps muscle strength was assessed using isokinetic equipment for five repetitions on both the left and right sides (LT and RT), following a 10-minute ankle joint warm-up performed by all participants. The extension (EX) strength and flexion (FL) strength of the quadriceps muscle were measured. Hand grip strength was measured three times for each hand alternately using a hydraulic hand dynamometer. To ensure the consistency of the results, all measurements for both indicators were conducted with participants maintaining a standardized posture. The quadriceps muscle quality index (MQI, Nm/kg) was calculated by dividing the EX and FL strength measurements (Nm) by the corresponding leg lean mass (kg) measured by dual-energy X-ray absorptiometry (DXA). MQI was calculated separately for the left and right sides, and the results were expressed as the change from baseline (Δpost-pre).

### DNA Extraction and Library Preparation

DNA was extracted from stool samples using the AccuStool DNA preparation kit (AccuGene) following the manufacturer’s protocol. The hypervariable V3–V4 region of the 16S rRNA gene was amplified using KAPA HiFi HotStart ReadyMix (Roche, Switzerland) and barcoded fusion primers (341F–805R) containing Nextera adaptor sequences, with a total of 25 PCR cycles. The resulting amplicons (approximately 420–470 bp) were purified using HIAccuBeads (AccuGene). Libraries were then pooled in equimolar concentrations and sequenced on the Illumina MiSeq platform using the MiSeq Reagent Kit v3 (600-cycle).

### Sequence Processing and Taxonomic Assignment

Raw paired-end sequencing reads generated using the Illumina MiSeq platform targeting the bacterial 16S rRNA gene V3–V4 region were denoised by correcting amplicon errors and used to infer exact amplicon sequence variants (ASVs) using DADA2 v1.16 [30]. Taxonomic classification was performed using Naïve Bayes classifiers trained on the SILVA release 138 rRNA reference database for V3–V4 sequences [31]. All downstream analyses of quality-filtered and chimera-filtered reads were conducted using the QIIME2-2024.5 software package [32].

### Fecal Microbiome Analysis

ASV (feature) tables, taxonomic assignments, and phylogenetic trees generated using the QIIME2 pipeline were imported into R (version 4.3.3). A phyloseq object was constructed using the phyloseq package by integrating feature abundance data, taxonomic information, sample metadata, and phylogenetic trees. Microbial abundance data were normalized using relative abundance (RA). To evaluate intervention-associated changes in gut microbiota composition, within-group comparisons of relative abundance were performed between baseline and endline within each group. In addition, to assess differences in intervention effects between groups, changes (Δpost-pre) were calculated for each participant and used for between-group comparisons. To assess microbial richness and evenness, multiple alpha diversity indices were calculated, including Observed features, Shannon, and Chao1. Beta diversity, reflecting differences in microbial community composition among samples, was assessed using Bray–Curtis distance. Principal Coordinate Analysis (PCoA) was performed based on each distance metric to visualize patterns of microbial community dissimilarity. Gut microbial functional profiles were predicted using PICRUSt2 (Phylogenetic Investigation of Communities by Reconstruction of Unobserved States 2) with reference to the Kyoto Encyclopedia of Genes and Genomes (KEGG) database. Predicted metabolic pathway enrichment profiles were subsequently normalized using centered log-ratio (CLR) transformation.

### Statistical Analysis

All results are presented as mean ± standard deviation (SD). Prior to statistical testing, normality of the data was assessed using the Shapiro–Wilk test, and appropriate statistical methods were selected based on the results of the normality assessment. For within-group comparisons between baseline and endline, paired t-tests were applied when data followed a normal distribution, while Wilcoxon signed-rank tests were used for non-normally distributed data. To evaluate between-group differences in intervention effects, changes (Δpost-pre) were calculated for each participant. Independent t-tests were used for normally distributed changes from baseline, whereas Mann–Whitney U tests were applied for non-normally distributed data. To assess associations between clinical parameters and gut microbiome composition, correlation analyses were performed between clinical data and microbial relative abundances using spearman’s rank correlation analysis. All statistical analyses were conducted using R software, and a *p*-value < 0.05 was considered statistically significant. *p*-values were adjusted for multiple comparisons using the Benjamini–Hochberg false discovery rate (FDR) method.

## Results

### Participant Characteristics

Among the 138 participants screened, 100 met the eligibility criteria and were randomly assigned to either the placebo group (*n* = 50) or the LPMYO group (*n* = 50). During the study period, 11 participants withdrew due to withdrawal of consent, violation of exclusion criteria, or use of contraindicated drugs. Consequently, a total of 89 participants who completed the 12-week intervention were included in the final analysis (placebo group: *n* = 45; LPMYO group: *n* = 44) (Fig. 2). The mean age of the participants was approximately 60 years, and both groups showed a compliance rate of over 90%. In addition, there were no significant differences between the groups in baseline characteristics, including sex, height, body weight, BMI, skeletal muscle mass, and blood pressure (Table 1).

### Clinical Effects of Heat-treated LPMYO

Quadriceps muscle strength, including EX and FL, as well as hand grip strength, was assessed on both the LT and RT during the 12-week intervention (Table 2). For EX, EX_LT significantly increased at endline compared with baseline only in the LPMYO group (*p* < 0.001), whereas no significant change was observed in the placebo group (*p* = 0.270). EX_RT significantly increased in both the LPMYO group (*p* < 0.001) and the placebo group (*p* < 0.05). For FL, significant increases were observed from baseline to endline on both sides only in the LPMYO group (FL_LT: *p* < 0.001; FL_RT: *p* < 0.01), whereas no significant changes were found in the placebo group (FL_LT: *p* = 0.759; FL_RT: *p* = 0.729). Hand grip increased significantly from baseline to endline in both groups bilaterally, with greater improvements in the LPMYO group (LT: LPMYO *p* < 0.001, placebo *p* < 0.01; RT: LPMYO *p* < 0.001, placebo *p* < 0.001). In addition, the between-group comparison of changes showed that both EX and hand grip increased significantly on both sides, whereas for FL, a significant difference was found for the FL_LT. To account for sex-related physiological differences, a subgroup analysis was conducted in male participants (Table S2). Consistent with the overall findings, significant improvements in quadriceps muscle strength and handgrip strength were observed following heat-treated LPMYO supplementation. These findings suggest that the beneficial effects of heat-treated LPMYO on muscle strength were generally consistent regardless of sex.

### Muscle Quality Index (MQI)

MQI was calculated as an indicator of muscle quality (Fig. 3). No significant within-group changes were observed in the placebo group for MQI, including EX_LT, EX_RT, FL_LT, and FL_RT (*p* > 0.05). In contrast, significant increases were observed in the LPMYO group for EX_LT (baseline vs. endline; 13.24 ± 3.91 vs. 15.38 ± 2.69, *p* < 0.0001), EX_RT (baseline vs. endline; 13.13 ± 3.75 vs. 14.81 ± 3.09, *p* < 0.0001), FL_LT (baseline vs. endline; 7.36 ± 2.13 vs. 8.36 ± 1.75, *p* < 0.0001), and FL_RT (baseline vs. endline; 7.26 ± 2.39 vs. 8.06 ± 1.67, *p* < 0.05) following supplementation. Furthermore, the changes from baseline were significantly greater in the LPMYO group than in the placebo group for EX_LT (LPMYO vs. PLA; 2.15 ± 2.44 vs. 0.03 ± 2.54, *p* < 0.001), EX_RT (LPMYO vs. PLA; 0.99 ± 1.46 vs. -0.11 ± 1.67, *p* < 0.001), FL_LT (LPMYO vs. PLA; 1.67 ± 2.29 vs. 0.32 ± 2.54, *p* < 0.05), and FL_RT (LPMYO vs. PLA; 0.80 ± 2.09 vs. -0.08 ± 1.18, *p* < 0.05).

### Effects on the Gut Microbiota Composition and Diversity

Changes in fecal microbial composition in each group are shown in Fig. 4. No significant changes were observed in alpha diversity or beta diversity between the groups after the intervention. However, several compositional differences in the gut microbiota were observed in the LPMYO group compared with the placebo group. At the family level, *Oxalobacteraceae* significantly increased, whereas *Peptococcaceae* significantly decreased in the LPMYO group after the intervention. At the genus level, *Faecalibacterium*, *Lachnospira*, [*Eubacterium*]_*ventriosum*_group, *Acidaminococcus*, *Leyella*, *Megasphaera*, and *Turicibacter* significantly increased in the LPMYO group (*p* < 0.05). In contrast, *Anaerobutyricum* significantly decreased and *Victivallis* significantly increased in the placebo group (*p* < 0.01). Furthermore, between-group comparisons showed significant differences in *Lachnospira*, [*Eubacterium*]_*ventriosum*_group, *Leyella*, *Turicibacter*, and *Victivallis* (*p* < 0.05).

### Correlation between Muscle Parameters and Gut Microbiota

Spearman’s correlation analysis revealed significant associations between gut microbiota and muscle-related parameters, including quadriceps muscle (EX and FL), and hand grip strength. At the family level, *Lachnospiraceae* and *Bacteroidaceae* showed positive correlations with hand grip strength, whereas *Methanomethylophilaceae* and *Defluviitaleaceae* were negatively correlated. For quadriceps muscle strength, *Bacteroidaceae* was positively correlated with EX_LT, and *Oxalobacteraceae* with FL_LT. *Rikenellaceae* showed significant positive correlations with bilateral quadriceps muscle strength as well as with MQI. In contrast, unclassified *Oscillospirales* exhibited consistent negative correlations across all measured parameters. At the genus level, *Bacteroides*, *Turicimonas*, and *Tyzzerella* were positively correlated with hand grip strength, while *Candidatus*_*Methanomethylophilus* showed a negative correlation. In addition, *Lachnospiraceae* NK4A136 group, *Turicimonas*, and *Megasphaera* were positively correlated with quadriceps muscle strength. In contrast, [*Clostridium*]_*innocuum*_group showed negative correlations with both hand grip strength and quadriceps muscle strength, and *Peptococcus* was negatively correlated with quadriceps flexion strength. Significant positive correlations were observed between MQI and the relative abundances of *Lachnospiraceae*_NK4A136_group, *Lachnospiraceae*_UCG-008, and *Turicimonas* (Fig. 5B). Importantly, analysis based on changes (Δpost-pre) demonstrated that *Peptococcaceae* was positively correlated with GRIP_RT, while *Turicibacter* was positively correlated with FL_RT and MQI_FL_RT (Fig. 5C, 5D).

### Predicted Gut Microbial Functional Profiles

Potential alterations in gut microbial metabolic pathways following heat-treated LPMYO supplementation were predicted using PICRUSt2. Functional prediction analysis identified significant alterations in metabolic pathways in the LPMYO group relative to the placebo group (Fig. 6). Notably, pathways involved in amino acid metabolism, including arginine biosynthesis, valine, leucine and isoleucine biosynthesis, cysteine and methionine metabolism, and alanine, aspartate and glutamate metabolism, were significantly enriched. Furthermore, pathways associated with cofactor and vitamin metabolism, energy metabolism, and carbohydrate metabolism were significantly upregulated following heat-treated LPMYO supplementation. Among these, nicotinate and nicotinamide metabolism, riboflavin metabolism, oxidative phosphorylation (OXPHOS), and the citrate (TCA) cycle exhibited significant increases after heat-treated LPMYO intake.

## Discussion

The present study was a 12-week randomized controlled trial designed to demonstrate the muscle strength-improving effect of heat-treated *Lactiplantibacillus plantarum* MYO (LPMYO) and to evaluate its impact on the gut microbiota in older adults with reduced muscle strength. Age-related declines in muscle strength are particularly evident in quadriceps muscle and hand grip strength [33-34]. Quadriceps muscle strength, commonly assessed by extension and flexion strength, is widely used as a representative indicator of lower-limb muscle strength [35]. In contrast, hand grip strength is a simple and widely accepted indicator of overall muscle strength and is commonly used in the diagnosis and assessment of sarcopenia [36]. In the present study, supplementation with heat-treated LPMYO significantly improved bilateral quadriceps muscle strength and hand grip strength compared with placebo in older adults with reduced muscle strength. Similarly, recent clinical studies investigating probiotics and paraprobiotics have reported beneficial effects on muscle strength and physical function in older adults. Lee *et al*. (2021) demonstrated that 18 weeks of supplementation with viable *L. plantarum* TWK10 improved hand grip strength, lower-limb muscle strength, and gait performance in older adults. In addition, heat-treated *L. paracasei* PS23 improved lower-limb muscle strength and endurance in older adults and was reported to exert greater effects than its viable counterpart. The growing body of literature supporting the muscle-strengthening effects of probiotics and paraprobiotics is consistent with our findings and suggests that heat-treated LPMYO may represent a promising paraprobiotic intervention for maintaining and improving muscle strength in older adults [37-38]. Although age-related muscle weakness has been attributed primarily to the loss of muscle mass, deterioration in muscle quality has recently emerged as another major contributor to age-related declines in muscle strength [39-40]. Muscle quality is defined as the amount of force generated per unit of muscle mass [41]. Previous studies have proposed the MQI as an indicator of muscle quality and have demonstrated that it exhibits higher sensitivity and reliability than other functional performance measures such as gait speed [42]. Importantly, the quadriceps MQI was significantly increased in the LPMYO group compared with the placebo group, suggesting that the muscle strength-enhancing effects of heat-treated LPMYO are linked to improvements in muscle quality. Likewise, our preclinical study also showed improvements in muscle strength such as grip strength and endurance capacity in heat-treated LPMYO treated mice [29]. In particular, heat-treated LPMYO suppressed the expression of muscle atrophy–related genes (Atrogin-1 and MuRF-1) induced by dexamethasone treatment, thereby restoring the aberrant expression of Myh4 (a marker gene of fast-twitch muscle fibers type II) to normal levels and eventually inducing positive alterations in muscle fiber composition. Skeletal muscle fibers are broadly classified into slow-twitch (type I) and fast-twitch (type II) fibers, and characteristic alterations in muscle fiber composition are commonly observed under muscle atrophy conditions [43-44]. Specifically, the distribution of these fiber types is considered one of the important indicators reflecting overall muscle quality [45-46]. According to previous studies, age-related declines in quadriceps muscle power and contraction velocity have been closely associated with quantitative and qualitative changes in fast-twitch (type II) muscle fibers [47-49]. Therefore, the muscle strength-enhancing effect of heat-treated LPMYO may be attributed to improvements in muscle quality mediated by the regulation of muscle fiber composition.

Recently, as the relationship between age-related muscle strength decline and the gut microbiota has been progressively elucidated, research interest in the gut–muscle axis has been increasing. However, clinical evidence supporting the gut–muscle axis remains limited. Therefore, it is important to investigate changes in the gut microbiota accompanying improvements in muscle strength and to explore the association between muscle strength enhancement and alterations in gut microbiota composition. Interestingly, heat-treated LPMYO supplementation not only significantly improved muscle strength by enhancing muscle quality, but also induced favorable changes in gut microbiota composition. At the family level, the relative abundance of *Oxalobacteraceae* significantly increased and that of *Peptococcaceae* significantly decreased in the LPMYO group compared with the placebo group, and these changes showed positive correlations with quadriceps flexion strength and hand grip strength. *Oxalobacteraceae* is known as a major bacterial family involved in intestinal oxalate metabolism [50]. When oxalate accumulates in the gut, it may contribute to muscle strength decline through impaired mitochondrial function, and therefore oxalate-degrading bacteria may play an important role in maintaining muscle health [51]. Indeed, Yang *et al*. (2024) reported that, in an aged mouse model, the reduction of oxalate following *Pisum sativum* sprout administration was associated with the alleviation of oxidative stress and muscle damage [52]. In contrast, *Peptococcaceae* is a bacterium associated with the induction of inflammation and endotoxin production, and the increase of such harmful bacteria during the aging process may contribute to muscle strength decline [53-55].

Favorable changes in the gut microbiota were prominently observed at the genus level. Compared with the placebo group, the LPMYO group showed significant increases in *Faecalibacterium*, *Lachnospira*, *Turicibacter*, *Megasphaera*, and [*Eubacterium*]_*ventriosum* group. In a mouse model, administration of ginseng-derived Rg3 increased hand grip strength along with an increase in intestinal *Eubacterium* *ventriosum* (EV), and EV has been reported as a key bacterial species that improves muscle health in mice with muscle atrophy [56]. In addition, *Faecalibacterium prausnitzii* EB-FPDK11 is associated with the amelioration of muscle atrophy through the promotion of mitochondrial biogenesis, the suppression of muscle atrophy-related gene expression, and anti-inflammatory action [57]. *Lachnospira* is a genus belonging to the family *Lachnospiraceae* and has been reported to show a positive correlation with hand grip strength, which supports the positive correlation between *Lachnospiraceae* and hand grip strength observed in the present study after heat-treated LPMYO supplementation [58]. Also, *Faecalibacterium*, *Lachnospira*, and [*Eubacterium*]_*ventriosum* group are known as beneficial bacteria that produce short-chain fatty acids (SCFAs), and SCFA-producing bacteria have drawn attention as key microbial indicators related to muscle strength in older adults [59-61]. Consistent with this hypothesis, older adults with reduced muscle strength have been reported to exhibit gut microbial dysbiosis along with decreased fecal butyrate concentrations compared with non-sarcopenic older adults [62]. Conversely, in older adults who maintained normal muscle strength, the abundance of SCFA-producing bacteria has been consistently reported to be high [63-65]. Indeed, *Lactobacillus casei* Shirota (LcS) has been reported to alleviate muscle strength decline and mitochondrial dysfunction in SAMP8 mice, and this effect has been reported to result from the restoration of SCFAs concentrations [66]. Most notably, *Megasphaera* was positively related to quadriceps muscle strength, whereas *Turicibacter* was related to improvements in right quadriceps flexion strength and the corresponding MQI. *Megasphaera* is a bacterial genus that produces SCFAs by utilizing lactate, and its reduction has been reported in previous studies to contribute to frailty in older adults [67-68]. Meanwhile, *Turicibacter* has been reported to improve skeletal muscle insulin sensitivity by mediating butyrate production and to enhance muscle strength in mouse models [69-70].

PICRUSt2-based functional prediction analysis revealed that pathways implicated in mitochondrial function and energy metabolism were enriched in the LPMYO group [71-72]. Recently, alterations in these metabolic pathways have been recognized as important biomarkers of sarcopenia [73]. In particular, amino acid metabolism, nicotinate and nicotinamide metabolism, riboflavin metabolism, and oxidative phosphorylation (OXPHOS) have been reported to be downregulated during age-related declines in muscle strength and are closely associated with the maintenance of muscle function [74-76]. Amino acid metabolism contributes not only to protein synthesis but also serves as a substrate for energy production, thereby supporting improvements in muscle mass and muscle quality [77-78]. In addition, nicotinate and nicotinamide metabolism and riboflavin metabolism are involved in the biosynthesis of essential cofactors for mitochondrial energy metabolism, including nicotinamide adenine dinucleotide (NAD^+^) and flavin adenine dinucleotide (FAD)/ flavin mononucleotide (FMN), respectively [79]. Age-related muscle weakness has been closely linked to mitochondrial dysfunction and reduced OXPHOS efficiency, which can ultimately impair energy production in skeletal muscle [80]. Previous studies have demonstrated that activation of these metabolic pathways contributes to a shift toward a more oxidative muscle fiber phenotype and improves muscle quality [81]. Therefore, the enrichment of these predicted metabolic pathways in the LPMYO group suggests that the gut microbiota may possess enhanced functional potential related to mitochondrial energy metabolism, which may be linked to improvements in muscle strength and muscle quality [82-83]. From this perspective, reduced abundance of *Lachnospira* and *Eubacterium*, together with decreased amino acid metabolism-related functions, has been reported in individuals with sarcopenia [65]. In addition, administration of *Faecalibacterium prausnitzii* to aged mice has been reported to be associated with enhanced TCA cycle, improved OXPHOS, an increased NAD^+^/NADH ratio, and enhanced mitochondrial function, and has been proposed as a key microbial taxon that may contribute to improvements in muscle health [84]. Moreover, *Turicibacter* has been associated with various metabolic pathways, including amino acid metabolism, as well as inflammatory responses, and may therefore contribute to the recovery from muscle damage and fatigue [85-86]. Based on these findings, we speculate that the metabolic characteristics of these gut microbes may have contributed to the observed enhancement in muscle strength and muscle quality.

Taken together, supplementation with heat-treated LPMYO significantly improved muscle quality and muscle strength in older adults with reduced muscle strength and induced favorable changes in the gut microbiota by promoting the proliferation of beneficial bacteria. In particular, several bacterial genera showed significant correlations with muscle strength indices, suggesting an association between changes in the abundance of specific gut microbial taxa and muscle strength-related parameters. Furthermore, functional prediction analysis revealed the enrichment of potential gut microbial metabolic pathways related to muscle health, including amino acid metabolism and OXPHOS. However, as this was an exploratory clinical study, further mechanistic studies investigating inflammatory responses and microbiota-derived metabolites, including SCFAs and amino acids, are needed. Furthermore, as the functional analysis was based on predictions derived from 16S rRNA gene sequencing data, future shotgun metagenomic and metabolomic analyses are needed to more clearly elucidate the observed functional changes. In addition, validation of mitochondrial function and skeletal muscle energy metabolism is required.

## Supplemental Materials

Supplementary data for this paper are available on-line only at http://jmb.or.kr.



## Figures and Tables

**Fig. 1:**
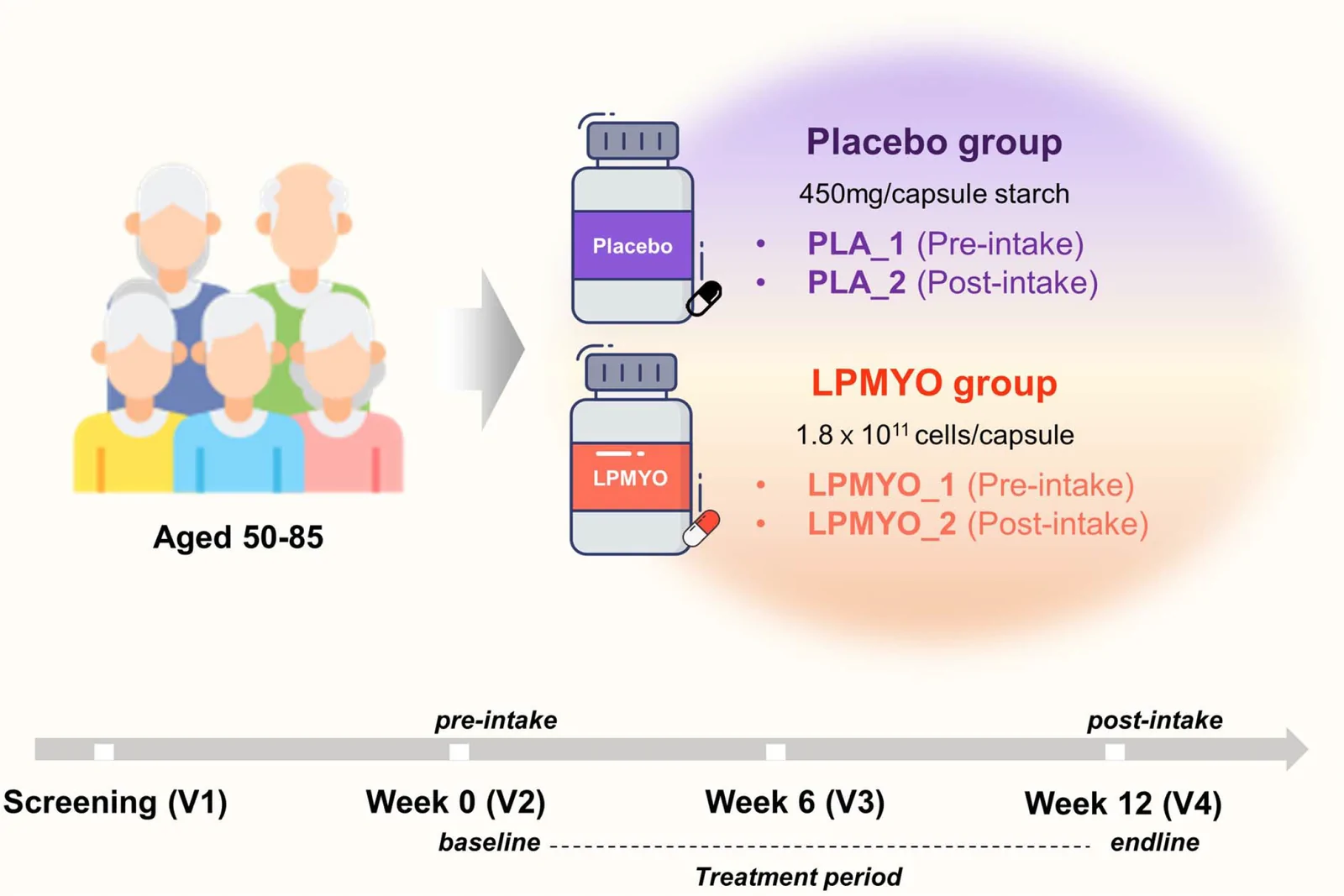
Experimental flowchart of LPMYO and placebo group.

**Fig. 2:**
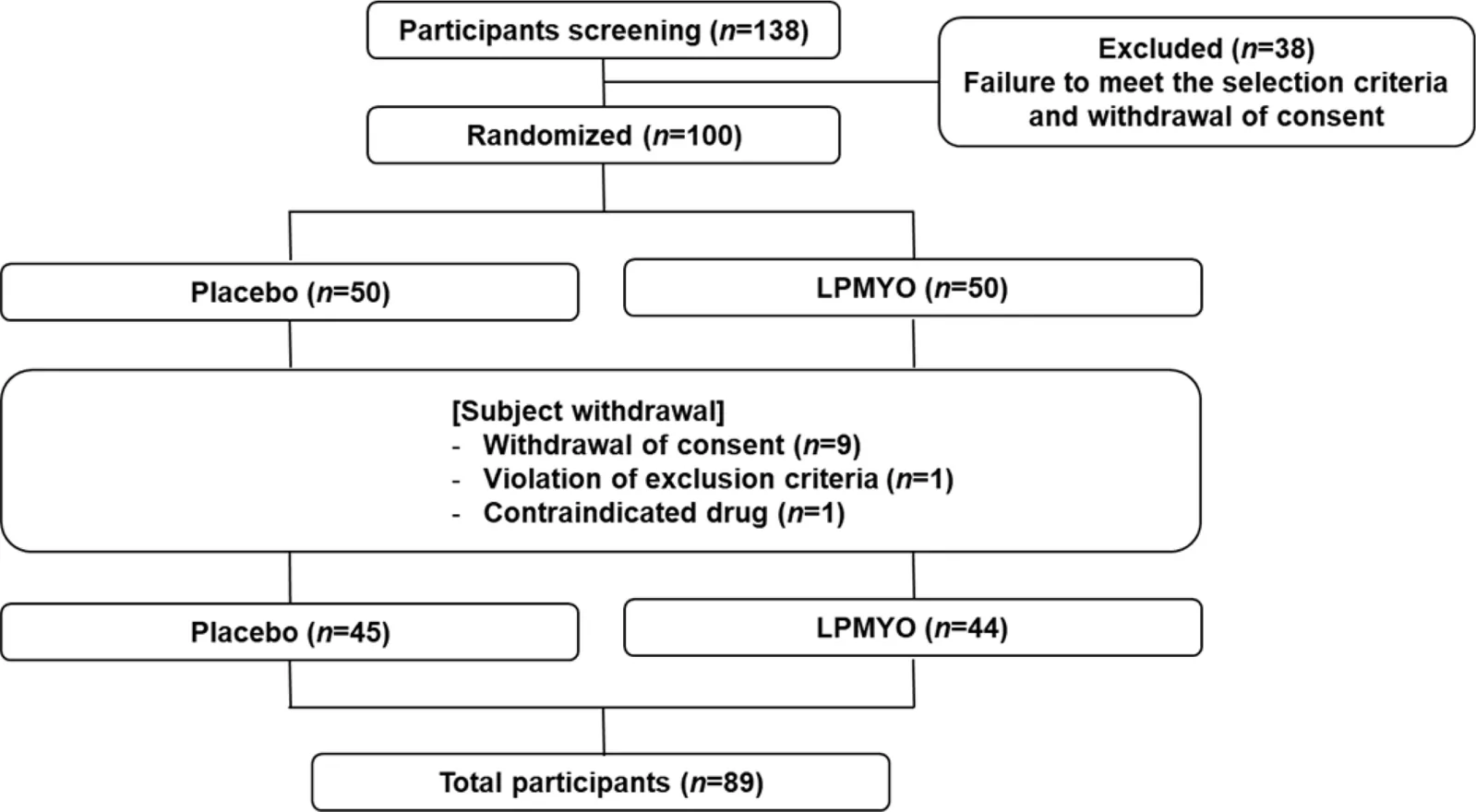
Participants flowchart of LPMYO and placebo group.

**Fig. 3:**
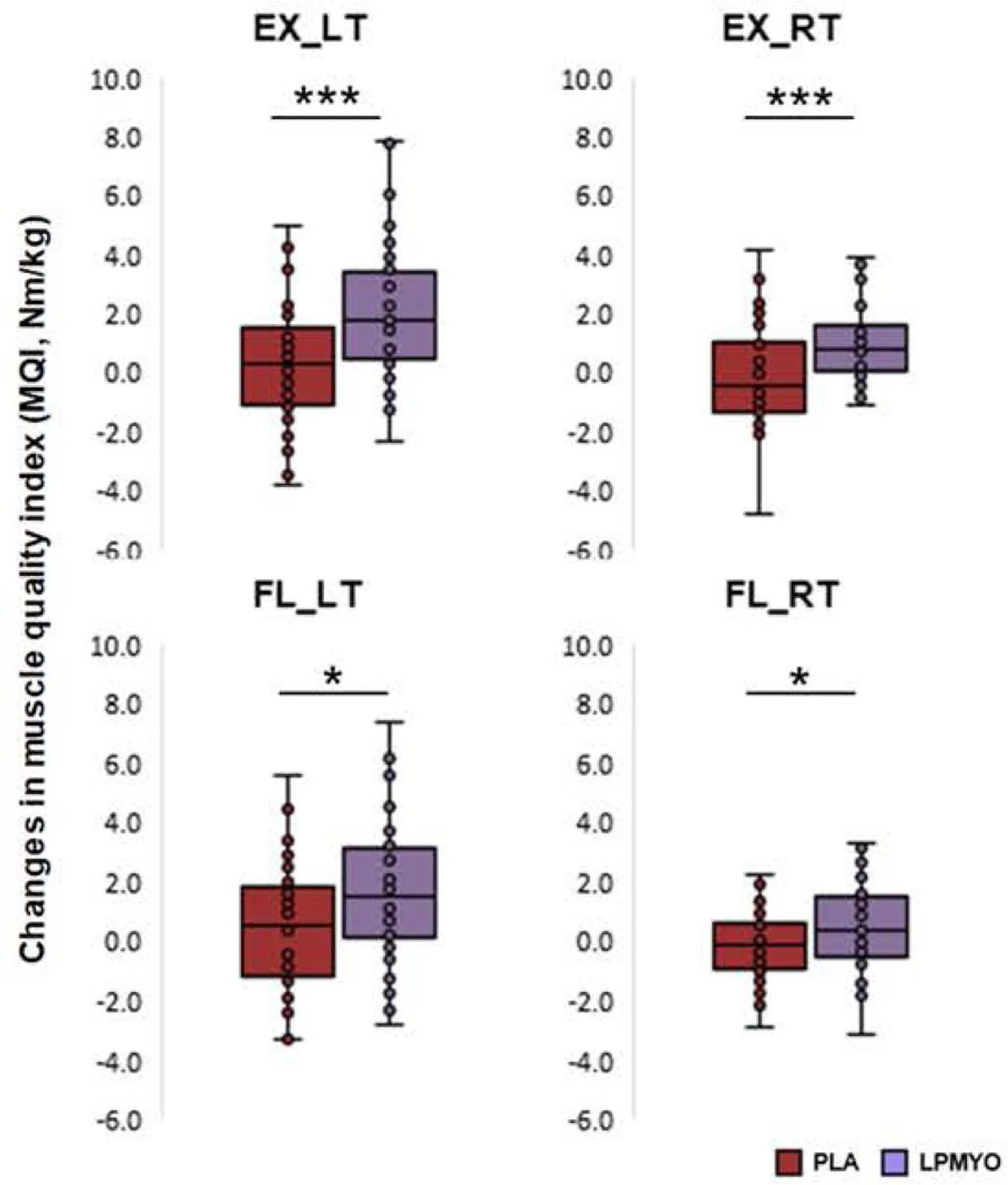
Changes in quadriceps muscle quality index (MQI) in the placebo (PLA) and LPMYO groups. MQI values were calculated for left (LT) and right (RT) quadriceps extension (EX) strength and left and right quadriceps flexion (FL) strength. *** *p* < 0.001, ** *p* < 0.01, * *p* < 0.05.

**Fig. 4:**
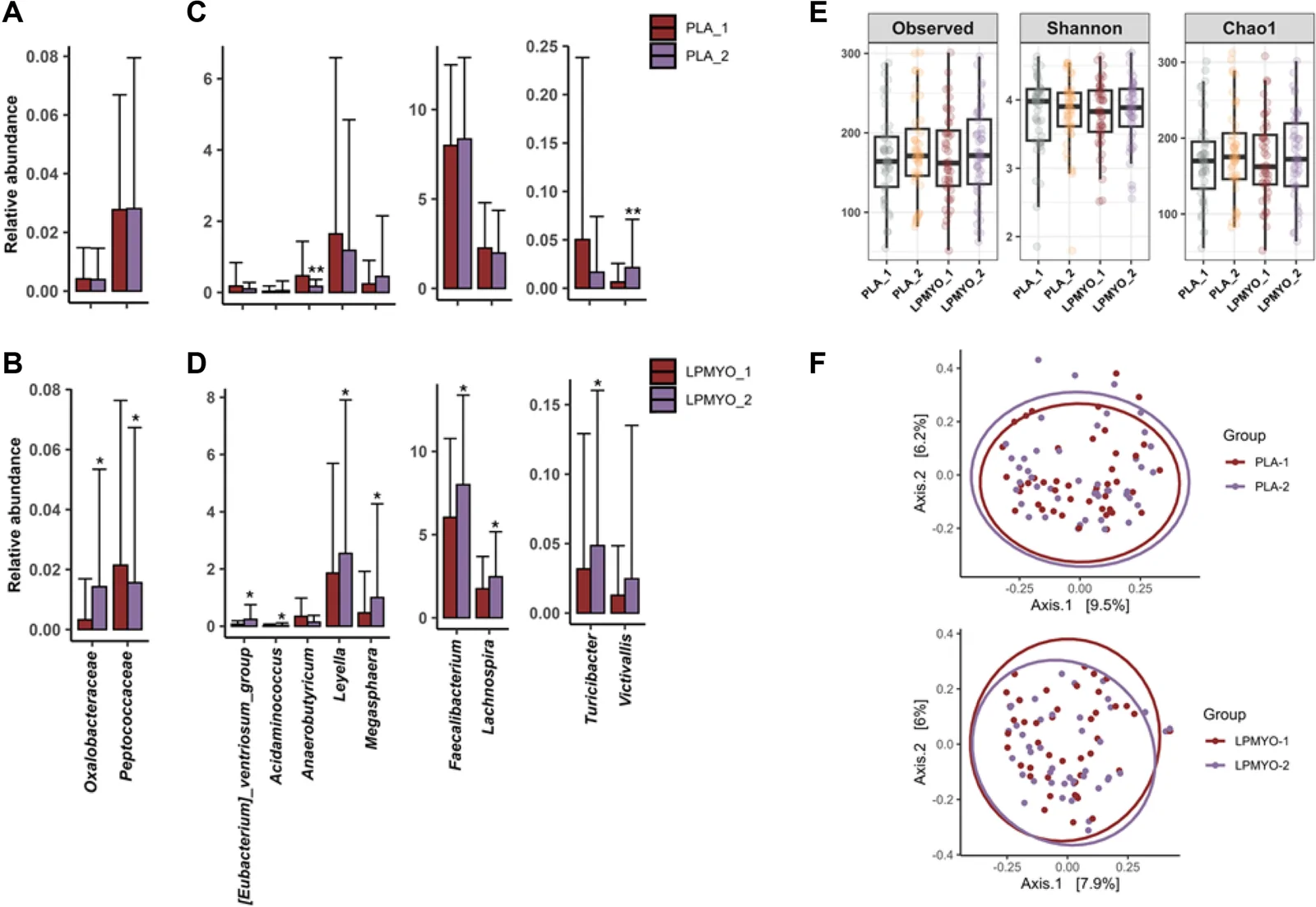
Effects of heat-treated LPMYO supplementation on gut microbiome composition and diversity. Relative abundance of microbial taxa at the family level in the (**A**) placebo (PLA) group and (**B**) LPMYO group. Relative abundance at the genus level in the (**C**) placebo group and (**D**) LPMYO group. (**E**) Alpha diversity indices, including Observed species, Shannon, and Chao1. (**F**) Beta diversity based on Bray–Curtis dissimilarity, visualized by principal coordinates analysis (PCoA). * *p* < 0.05, ** *p* < 0.01.

**Fig. 5:**
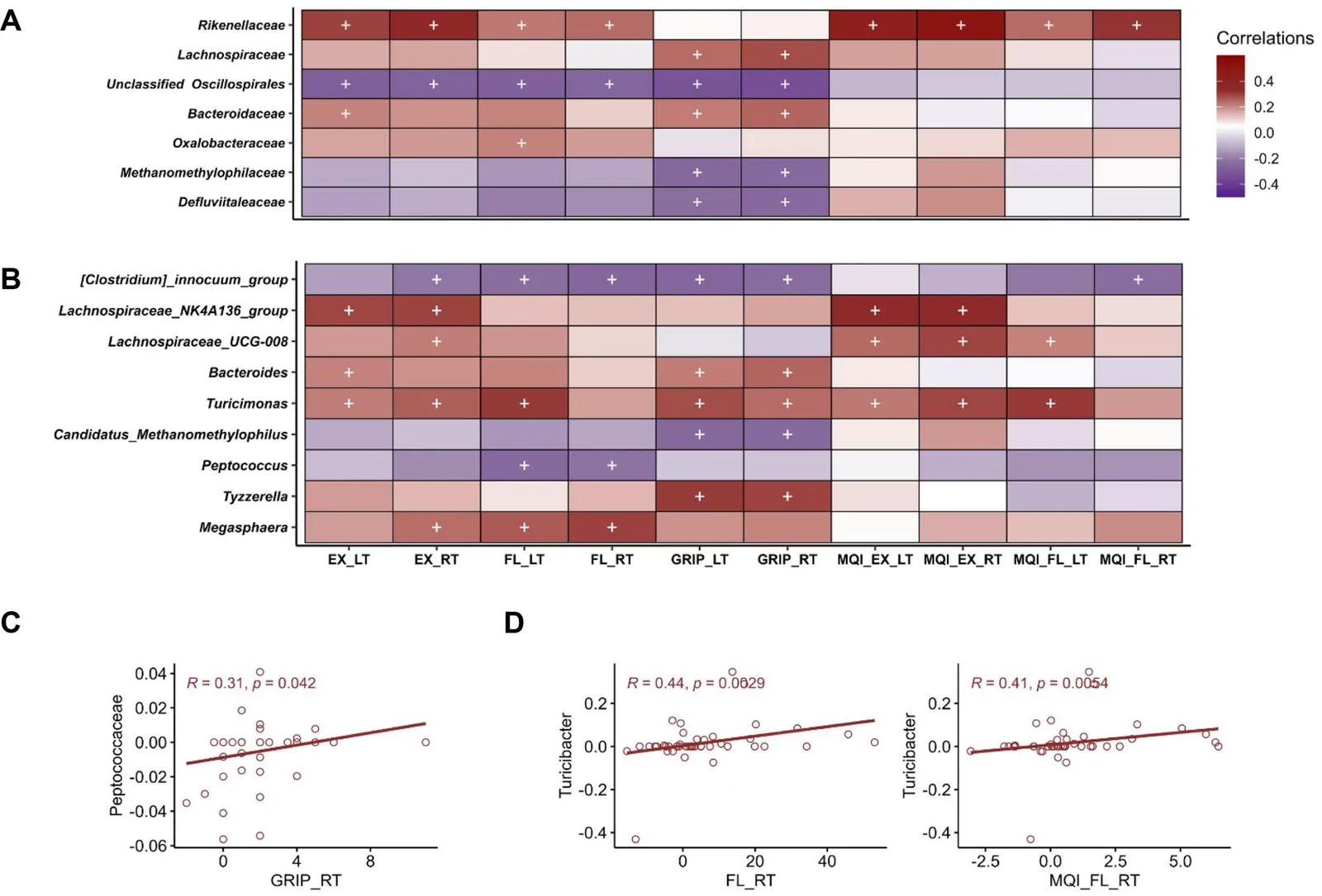
Correlation analysis between muscle strength parameters and gut microbiome composition. Correlations between relative abundance and muscle strength parameters at the (**A**) family and (**B**) genus levels. Correlations between changes (Δpost-pre) in relative abundance and changes in muscle strength parameters at the (**C**) family and (**D**) genus levels. Muscle strength parameters included left (LT) and right (RT) quadriceps extension (EX) strength, left and right quadriceps flexion (FL) strength, bilateral hand grip (GRIP) strength, and the muscle quality index (MQI) for left and right quadriceps extension and flexion strength. + *p* < 0.05.

**Fig. 6:**
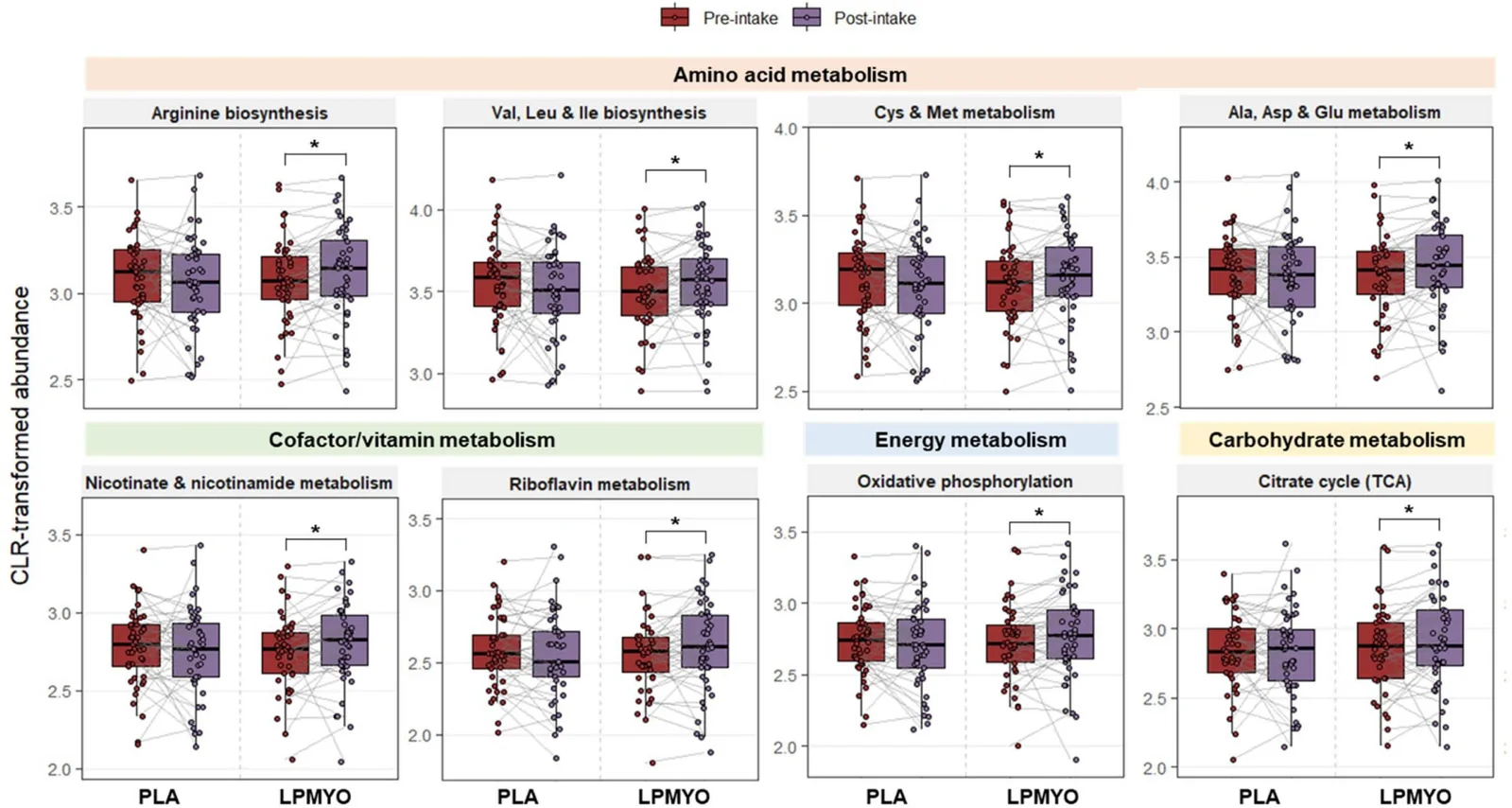
PICRUSt2-predicted microbial metabolic pathways significantly altered following the intervention. Relative changes in KEGG metabolic pathways between the placebo (PLA) and LPMYO groups are shown. Pathways exhibiting significant differences included arginine biosynthesis, valine, leucine and isoleucine biosynthesis (Val, Leu & Ile), cysteine and methionine (Cys & Met) metabolism, alanine, aspartate and glutamate (Ala, Asp & Glu) metabolism, nicotinate and nicotinamide metabolism, riboflavin metabolism, oxidative phosphorylation (OXPHOS), and the citrate (TCA) cycle. * *p* < 0.05.

**Table 1:** Baseline characteristics of the participants.

	Placebo (*n*=45)	LPMYO (*n*=44)	*p*-value
Age	59.31±9.71	61.09±8.11	0.351
Gender (Female)	11	12	0.761
Gender (Male)	34	32	
Height (cm)	168.48±7.94	165.85±7.24	0.106
Body Weight (kg)	64.13±8.87	63.30±9.18	0.665
BMI (kg/m^2^)	22.55±2.40	22.94±2.30	0.432
Skeletal Muscle Mass (kg)	25.88±4.35	25.26±4.31	0.502
Systolic blood pressure (mmHg)	124.24±11.38	126.70±12.65	0.337
Diastolic blood pressure (mmHg)	75.11±8.67	73.80±8.17	0.464
Adherence (%)	96.00±4.46	94.94±5.28	0.310

**Table 2:** Changes in quadriceps muscle strength and hand grip strength after 12 weeks of supplementation in the placebo and LPMYO groups.

		Placebo (*n*=45)				LPMYO (*n*=44)				*p*-value^3^
		Pre-intake	Post-intake	*p*-value^1^	Δpost-pre	Pre-intake	Post-intake	*p*-value^2^	Δpost-pre	
EX (Nm)	LT	97.58 ± 27.67	99.67 ± 24.01	0.270	2.10 ± 12.58	89.18 ± 31.95	104.24 ± 26.76	<0.001	15.06 ± 16.42	<0.001
	RT	100.48 ± 28.75	105.10 ± 27.62	<0.05	4.62 ± 13.64	94.43 ± 32.45	106.63 ± 29.81	<0.001	12.21 ± 16.09	<0.05
FL (Nm)	LT	52.60 ± 15.86	53.06 ± 14.36	0.759	0.46 ± 10.03	49.14 ± 16.51	56.38 ± 14.72	<0.001	7.24 ± 9.10	<0.01
	RT	51.33 ± 15.79	51.75 ± 15.13	0.729	0.42 ± 8.12	51.97 ± 19.50	57.98 ± 15.73	<0.01	6.02 ± 14.55	0.088
GRIP (kg)	LT	30.03 ± 4.67	30.67 ± 4.89	<0.01	0.63 ± 1.56	29.69 ± 5.34	31.20 ± 5.74	<0.001	1.51 ± 1.85	<0.05
	RT	30.27 ± 4.94	31.00 ± 4.81	<0.001	0.73 ± 1.23	30.59 ± 5.56	32.23 ± 5.73	<0.001	1.64 ± 2.23	<0.05

Quadriceps extension (EX) strength, Quadriceps flexion (FL) strength, hand grip (GRIP) strength, Left (LT), and Right (RT)

^1^Compared within placebo groups; analyzed by paired t-test or Wilcoxon signed rank test

^2^Compared within LPMYO groups; analyzed by paired t-test or Wilcoxon signed rank test

^3^Compared between groups; analyzed by independent t-test or Mann-Whitney U test
