# Supplementary material for: Heat-treated Lactiplantibacillus plantarum MYO Improves Muscle Strength and Modulates Gut Microbiota in Older Adults with Reduced Muscle Strength
Source: J Microbiol Biotechnol. 2026 Oct 7;36:e2607020. doi: 10.4014/jmb.2607.07020 (PMC13686804; doi:10.4014/jmb.2607.07020)
Supplement: Supplementary file 1 [file jmb-36-e2607020-supple.pdf]

J. Microbiol. Biotechnol. 2026. 36: e2607020

https://doi.org/10.4014/jmb.2607.07020

Supplementary Tables

Table S1. Detailed Included and Excluded criteria.

Included criteria	Excluded criteria
(1) Adults aged ≥50 and ≤85 years at the time of screening, with handgrip strength below the standard reference values* but not meeting the criteria for sarcopenia<br>(i.e., handgrip strength ≤28kg for men and ≤18kg for women according to the 2019 Asian Working Group for Sarcopenia guidelines) *Based on the 2022 Korea National Health Statistics	(1) A total score of ≥4 on the SARC-F questionnaire
(2) Individuals with skeletal muscle mass (SMM) less than 100% of the standard reference range	(2) Individuals with any of the following conditions:<br>▪ Diagnosed sarcopenia or a history of fragility hip fracture<br>▪ Musculoskeletal disorders or injuries affecting gait (e.g., rheumatoid arthritis requiring treatment, spinal stenosis with claudication, severe osteoarthritis, spinal cord injury)<br>▪ Psychiatric disorders requiring medication (except for intermittent medication for sleep disorders)<br>▪ History of malignancy within the past 5 years or currently undergoing cancer treatment<br>▪ Severe dementia or Parkinson's disease impairing communication or ambulation (mild cases may be included)<br>▪ Alcohol dependence or suspected substance abuse<br>▪ Cardiovascular events within the past 3 months, including myocardial infarction, coronary artery bypass grafting, percutaneous coronary intervention (e.g., angioplasty or stent
(3) Individuals who voluntarily agree to participate in the study, fully understand the study procedures, and are capable of complying with all study requirements	

insertion), unstable angina, New York Heart Association Class III–IV heart failure, severe valvular heart disease (e.g., aortic or mitral stenosis), stroke, or untreated atrial fibrillation

▪ Uncontrolled diabetes mellitus (HbA1c > 7.0%)

▪ Uncontrolled hypertension (SBP > 140mmHg or DBP > 90mmHg)

▪ History of pulmonary disease or systemic autoimmune disorders

(3) Use of the following medications, functional foods, or dietary supplements prior to screening:

▪ Use of medications related to muscle strength improvement within the past 3 months

▪ Intake of supplements or foods associated with muscle strength improvement (e.g., amino acids, creatine, protein supplements, ergogenic aids, hormone supplements, vitamin D) within the past 3 months
(Participants may be included if vitamin D intake has been maintained without dose change for more than 3 months)

▪ Use of oral steroids or hormone therapies (e.g., testosterone, estrogen, androgen, growth hormone, corticosteroids) within the past 4 weeks

▪ Continuous intake of fermented milk or probiotic products within the past 4 weeks

▪ Intake of probiotics, prebiotics, or synbiotics within the past 2 weeks

▪ Use of antibiotics within the past 2 weeks

(4) Participation in regular aerobic or resistance exercise programs within the past 3 months

(5) Pregnant or breastfeeding women, or those planning pregnancy

(6) Excessive alcohol consumption (≥40g/day for men, ≥20g/day for women, based on WHO guidelines)

	(7)	Heavy smokers (≥20cigarettes/day)
	(8)	Participation in another clinical trial within the past 3 months
	(9)	Known allergy or hypersensitivity to the investigational product
	(10)	Planned participation in another clinical trial during the study per iod
	(11)	Abnormal laboratory findings at screening, including:<br>▪ eGFR < 60mL/min/1.73 m^2<br>▪ ALT or AST > 3× upper limit of normal<br>▪ Creatinine > 2.0mg/dL<br>▪ Thyroid-stimulating hormone (TSH) < 0.1µIU/mL or > 10µIU/mL
	(12)	Any other condition that, in the opinion of the investigator, make s the participant unsuitable for study participation

Table 2. Changes in quadriceps muscle strength and hand grip strength in male participants of the placebo and LPMYO groups.

		Pre-intake	Post-intake	*p*-value[1]	Δpost-pre	Pre-intake	Post-intake	*p*-value[2]	Δpost-pre	*p*-value[3]
		**Placebo (*n*=34)**				**LPMYO (*n*=32)**				
**EX (Nm)**	**LT**	104.70 ± 27.26	106.41 ± 23.54	0.441	1.71 ± 12.81	101.66 ± 25.96	114.95 ± 22.93	<0.001	13.29 ± 15.55	<0.01
	**RT**	107.93 ± 27.99	112.22 ± 27.41	0.096	4.29 ± 14.60	107.36 ± 25.32	118.03 ± 26.35	<0.001	10.67 ± 15.73	0.09
**FL (Nm)**	**LT**	56.39 ± 15.94	57.45 ± 13.56	0.952	1.05 ± 11.17	54.32 ± 15.20	61.22 ± 13.54	<0.001	6.91 ± 8.40	<0.05
	**RT**	55.81 ± 15.25	56.33 ± 14.02	0.744	0.51 ± 9.07	57.12 ± 18.56	63.44 ± 14.34	<0.05	6.33 ± 14.12	<0.05
**GRIP (kg)**	**LT**	32.19 ± 3.03	32.94 ± 3.07	<0.05	0.75 ± 1.72	32.45 ± 3.11	34.11 ± 3.57	<0.001	1.66 ± 2.00	0.05
	**RT**	32.56 ± 3.13	33.18 ± 3.16	<0.01	0.62 ± 0.99	33.47 ± 3.23	35.08 ± 3.67	<0.001	1.61 ± 2.41	<0.05

Quadriceps extension (EX) strength, Quadriceps flexion (FL) strength, handgrip (GRIP) strength, Left (LT), and Right (RT)

[1] Compared within placebo groups; analyzed by paired t-test or Wilcoxon signed rank test

[2] Compared within LPMYO groups; analyzed by paired t-test or Wilcoxon signed rank test

[3] Compared between groups; analyzed by independent t-test or Mann-Whitney U test
